# Bictegravir/Emtricitabine/Tenofovir Alafenamide in Adults with HIV/HBV Coinfection: An Open-Label, Single-Arm, Safety and Efficacy Switch Study

**DOI:** 10.3390/v17040510

**Published:** 2025-03-31

**Authors:** Helena Kwakwa, Jacqueline Bran, Julia Ruff, Salma Sharaf, Hyunuk Seung, Sunny Choe, Joel V. Chua

**Affiliations:** 1 Newlands Health, Philadelphia, PA 19114, USA; hkwakwa@aol.com (H.K.); msjekingus@yahoo.com (J.R.); 2 Institute of Human Virology, University of Maryland School of Medicine, Baltimore, MD 21201, USA; jbran@som.umaryland.edu (J.B.); ssharaf@ihv.umaryland.edu (S.S.); 3 Department of Practice, Sciences, and Health Outcomes Research, University of Maryland School of Pharmacy, Baltimore, MD 21201, USA; hseung@rx.umaryland.edu; 4 Gilead Sciences, Inc., Foster City, CA 94404, USA; sunny.choe@gilead.com

**Keywords:** HIV, hepatitis B, HIV-HBV coinfection, bictegravir, tenofovir alafenamide

## Abstract

Background: HIV and hepatitis B virus (HBV) coinfection has been associated with a higher risk of morbidity and mortality. HBV-active antiretroviral regimens have significantly improved the outcomes for coinfected people. Although bictegravir/emtricitabine/tenofovir alafenamide (BIC/FTC/TAF) is safe and efficacious for the treatment of HIV, there are few randomized studies on the treatment of HIV/HBV coinfection. Methods: This open-label switch study enrolled adults with HIV/HBV coinfection from two clinical centers. The participants were switched from their current antiretroviral regimen (regardless of viral suppression) to BIC/FTC/TAF, taken once daily for 48 weeks. The primary endpoints were the proportion of participants with HIV RNA < 50 copies/mL and HBV DNA < 29 IU/mL at Week 24. Results: Twenty-eight participants were enrolled, with a median age of 51 years; the majority were Black (89%) and male (86%). At baseline, 71% (20/28) and 79% (22/28) were HIV- and HBV-suppressed, respectively, and 64% (18/28) exhibited suppression for both. At week 24, 89% (25/28) and 86% (24/28) were HIV- and HBV-suppressed, respectively, and 82% (23/28) exhibited suppression for both. The most common treatment-related adverse event was nausea (2/28). None of the participants discontinued the treatment due to an adverse event. No serious adverse events or hepatitis flares were observed. Conclusion: BIC/FTC/TAF is a safe and suitable option for the treatment of HIV/HBV-coinfected patients.

## 1. Introduction

HIV-1 is a life-threatening and serious disease of global public health importance; close to 40 million people worldwide have HIV-1 [1]. Of these individuals, about 5–20% are also coinfected with hepatitis B virus (HBV) [2,3]. HIV/HBV coinfection has been associated with a higher risk of morbidity and mortality, especially with regard to accelerated liver disease progression leading to hepatic failure, the need for liver transplantation, and death [4,5]. Historically, HBV coinfection accelerates the immunological and clinical progression of HIV infection and increases the risk of hepatotoxicity when combination antiretroviral treatment (ART) is initiated, while HIV infection increases the risk of hepatitis events, cirrhosis, and end-stage liver disease related to chronic HBV infection [6]. However, the availability of HBV-active ART regimens has significantly improved outcomes for the HIV/HBV coinfected population, leading to increased life expectancies and lower morbidity.

Bictegravir/emtricitabine/tenofovir alafenamide (BIC/FTC/TAF) is a fixed-dose combination ART regimen that is FDA-approved for the treatment of HIV-1 in children and adults. BIC/FTC/TAF contains an HIV-1 integrase strand transfer inhibitor, bictegravir (BIC), and two HIV-1 nucleoside/nucleotide analog reverse transcriptase inhibitors, tenofovir alafenamide (TAF) and emtricitabine (FTC). Both TAF and FTC exhibit activity against HBV [7]. BIC/FTC/TAF was shown to be highly effective in the initial treatment of HIV-1 infection in two phase-3 treatment-naïve trials: Studies 1489 and 1490 [8,9]. In the first trial (Study 1489), BIC/FTC/TAF demonstrated non-inferiority to DTG/ABC/3TC, but this excluded HIV/HBV-coinfected patients [9]. In Study 1490, BIC/FTC/TAF demonstrated non-inferiority to DTG + FTC/TAF [8]. There was no treatment-emergent resistance in either arm. In contrast to the previous study, people with HBV coinfection were eligible to enroll in Study 1490. Nevertheless, of the 320 patients in the study who received BIC/FTC/TAF, only 8 (2%) were coinfected with HBV; all eight of these HIV-HBV participants achieved viral suppression against both viruses [8].

Although the safety and efficacy of BIC/FTC/TAF have been well established for the treatment of HIV-1 infection and TAF has been found to be as efficacious and durable as TDF in the treatment of HBV infection, until recently, there have been limited data on the application of BIC/FTC/TAF to the HIV/HBV coinfected population, especially among Black adults. The results of ALLIANCE [10], the only randomized study to investigate whether BIC/FTC/TAF is non-inferior to dolutegravir plus emtricitabine and tenofovir disoproxil fumarate (DTG + FTC/TDF) for the suppression of HIV-1 RNA and HBV DNA for people with HIV/HBV coinfection starting antiviral therapy, were published recently. This study enrolled 243 treatment-naïve individuals, of whom only 8 were Black, and demonstrated that BIC/FTC/TAF was non-inferior to DTG + FTC/TDF for the suppression of HIV-1 (RNA < 50 copies/mL) and superior for the suppression of HBV (DNA < 29 IU/mL) at 48 weeks [10]. The two phase III registrational studies (treatment-naïve Study 1490 and switch Study 1878) that allowed HIV/HBV coinfected participants to be enrolled included a total of only 16 coinfected patients who received BIC/FTC/TAF [8,9,11]. All 16 had HIV-1 RNA < 50 copies/mL and HBV DNA < 29 IU/mL at Week 48 [11]. Likewise, in Study 1878, all eight volunteers with HIV/HBV coinfection maintained suppression against both viruses [11]. Thus, there is a need for additional data, especially regarding Black adults, to further validate the safety and efficacy of switching to BIC/FTC/TAF as a mainstay regimen for the treatment of people with HIV/HBV coinfection. This study was conducted to obtain real-world data to help address this need.

## 2. Materials and Methods

### 2.1. Study Design and Selection of Participants

We conducted an open label, single-arm, phase 4 switch study to evaluate the efficacy, safety, and tolerability of treatment with BIC/FTC/TAF among adults with HIV/HBV coinfection from May 2019 to November 2022 (ClinicalTrials.gov: NCT03797014). We enrolled adults coinfected with HIV and HBV from two clinical research centers in the United States (in Baltimore, Maryland, and Philadelphia, Pennsylvania). Eligible participants were at least 18 years of age with documented HIV-1 infection and chronic HBV infection and on a stable ART regimen for at least three months prior to enrollment, regardless of HIV or HBV suppression at the time of enrollment. Key exclusion criteria included having known allergies to any components of BIC/FTC/TAF, nursing or being pregnant, having tuberculosis, having an active malignancy or opportunistic illness, or having decompensated cirrhosis. Participants with known resistance to any components of BIC/FTC/TAF, except for M184V/I mutations, were also excluded from this study.

Written informed consent was obtained from all participants. This study was approved by the Institutional Review Board of both the University of Maryland (for the Baltimore site) and the Philadelphia Department of Health (for the Philadelphia site) and conducted in compliance with Good Clinical Practice guidelines, the Declaration of Helsinki, and regulatory requirements. An independent safety-monitoring committee reviewed the progress of this study.

### 2.2. Procedures

Regardless of HIV or HBV suppression, participants were switched from their current ART regimens to BIC/FTC/TAF (50/200/25 mg), administered orally once daily and without regard to food for 48 weeks. Safety evaluations consisted of adverse event (AE) monitoring, physical examination (including vital signs), and acquisition of clinical laboratory data. Plasma HIV-1 RNA and HBV DNA real-time PCR assays were used for efficacy assessments. Plasma HIV-1 RNA concentrations were measured using Cobas AmpliPrep/Cobas TaqMan HIV-1 Test version 2.0 (Roche Diagnostics, Indianapolis, IN, USA), with a lower limit of detection (LLOQ) of 20 copies/mL; plasma HBV DNA concentrations were measured using Cobas HBV Test for use on Cobas 6800/8800 system (Roche Diagnostics, Indianapolis, IN, USA), with an LLOQ of 10 IU/mL. Both safety and efficacy assessments were conducted at screening, on Day 1, and in Weeks 4, 8, 12, 24, 36, and 48. In addition, CD4 count and percentage were measured at screening, on Day 1, and in Weeks 12, 24, and 48. Serological markers that included hepatitis B surface antigen (HBsAg), e antigen (HBeAg), anti-hepatitis B core total antibody (anti-HBc), anti-hepatitis B surface antibodies (anti-HBs), and anti-hepatitis B e antibody (anti-HBe) were measured using commercially available assays at screening and in Weeks 24 and 48. As part of the exploratory evaluation, Fibroscan (transient elastography) tests were performed on Day 1 and in Weeks 24 and 48 for participants at the Baltimore site only.

### 2.3. Outcomes and Endpoints

Primary efficacy endpoints were the proportion of participants exhibiting HIV suppression (defined as HIV RNA < 50 copies/mL via FDA Snapshot method) and HBV suppression (defined as HBV DNA < 29 IU/mL via missing = failure method) at Week 24. The secondary endpoints included HIV and HBV suppression in Week 48, the proportion of participants who stopped taking the study medications due to any AE, changes from baseline in CD4 cell count and percentage, Fibroscan test score, and the proportion of individuals who exhibited alanine aminotransferase (ALT) normalization and HBsAg and/or HBeAg loss with or without anti-HBs or anti-HBe seroconversion.

### 2.4. Statistical Analyses

The primary analysis endpoint for evaluating treatment efficacy used to compute the sample size was the observed HIV-1 viral suppression rate. A true suppression rate in Week 24 of at least 91% would be considered active in this population. An exact binomial test with a type I error rate of 0.05 and a sample size of 60 would have 84% power for detecting the difference between the null-hypothesis proportion (number exhibiting suppression/total number treated) of 0.773 (based on published results) [12,13] and the research hypothesis proportion of 0.910 (our educated guess for the desired rate of success).

In the efficacy analyses, we employed both intent-to-treat (ITT) and per-protocol (PP) populations. The ITT population included all participants enrolled in the study who received at least one dose of the drug understudy (BIC/FTC/TAF), whereas the PP population included all participants who received at least one dose of BIC/FTC/TAF, had not committed any major protocol violations, and remained on study at all analysis timepoints (Week 24 for primary and Week 48 for secondary).

All safety analyses were performed using the ITT population. For each participant, safety data were collected, starting from the date that the drug being studied was first administered to the final day of participation in the study, and summarized in the safety analysis. The primary safety endpoint was the proportion of participants who discontinued the drug due to an AE.

The exact McNemar test was performed to compare the proportion of change in the discordant category between Day 0 and Week 24 (primary efficacy endpoints) and between Day 0 and Week 48 (Secondary efficacy endpoints). The estimated proportion of change and 95% confidence interval were computed using the exact binomial test. A nonlinear mixed model (NLMM) was used to analyze how the odds of outcomes changed over three time points (Day 0, Week 24, and Week 48). Analyses were conducted using R version 4.3.3 and SAS version 9.4 (SAS Institute, Cary, NC, USA).

## 3. Results

Of the 33 participants who provided consent and were screened, 28 met the eligibility criteria and were enrolled, and followed from May 2019 to December 2021 (Supplemental Figure S1). Twenty-five of the twenty-eight (89%) enrolled participants completed the 48-week study. None of them discontinued the therapy due to any AEs or virologic failure. Three discontinued participation due to other reasons: one was dismissed due to noncompliance, one was lost to follow-up, and one withdrew from the study. We were unable to accrue planned enrollment; as such, the results may be underpowered.

### 3.1. Baseline Characteristics

The 28 enrolled participants had a mean age of 50.1 (median 51.0) years, with a range of 34 to 71 years. The majority were Black (25/28; 89%), while 14% (4/28) were female, and 100% (28/28) were non-Hispanic. Regarding HIV, 71% (20/28) had baseline HIV-1 RNA < 50 copies/mL, with 68% (19/28) reporting HIV suppression for >12 months prior to enrollment. For HBV, 79% (22/28) had HBV DNA < 29 IU/mL, 48% (12/25) were HBeAg-positive, and 40% (10/25) had detectable anti-HBe antibodies at baseline. Five participants (18%) were found to be positive for hepatitis C (HCV) antibody, but they were all HCV RNA-negative when screened. Additionally, 18% (5/28) tested positive for hepatitis D (HDV) antibodies when screened, and 3 of these 5 were also HCV-antibody-positive. Three participants had no HBeAg or antibody data available. A total of 14% (4/28) had elevated baseline ALT levels ranging from 56 to 70 IU/L (normal value: ≤44 IU/L)—although none of the four had detectable HBV DNA levels at baseline (Table 1).

The mean baseline CD4 cell count was 531 cells/μL (median: 528, range: 155–1440, interquartile range [IQR]: 349–714), and the mean baseline CD4 percentage was 30.1% (median: 30.1, range: 17.5–54.7, IQR: 38.1–23.5). Only one participant had a CD4 count below 200 cells/μL at baseline.

FibroScan tests were conducted only for participants at the Baltimore site (n = 21). The mean fibrosis score at baseline was 9.17 kPa (median: 5.1, range: 2.7–75, IQR: 4.7–7.5). The majority had fibrosis scores of F0–F1 (score: ≤8 kPa; 15/21, 71%), followed by F1–F2 (score: 8–9 kPa; 3/21, 14%) and F3 (score: 9–11 kPa; 2/3, 10%). One participant had a baseline fibrosis score of F4 (75 kPa); he had normal ALT levels, was HBeAg-positive, and tested negative for both HCV and HDV.

Prior to enrollment in the study, 79% (22/28) of the participants were on a tenofovir-containing regimen, 19 of whom were on TAF. The six participants not on tenofovir at baseline were not on standard-of-care regimens for their HBV treatment—although four of the six had HBV DNA levels below the limit of detection at baseline (Supplementary Table S1).

### 3.2. Efficacy Results

**Figure 1:**
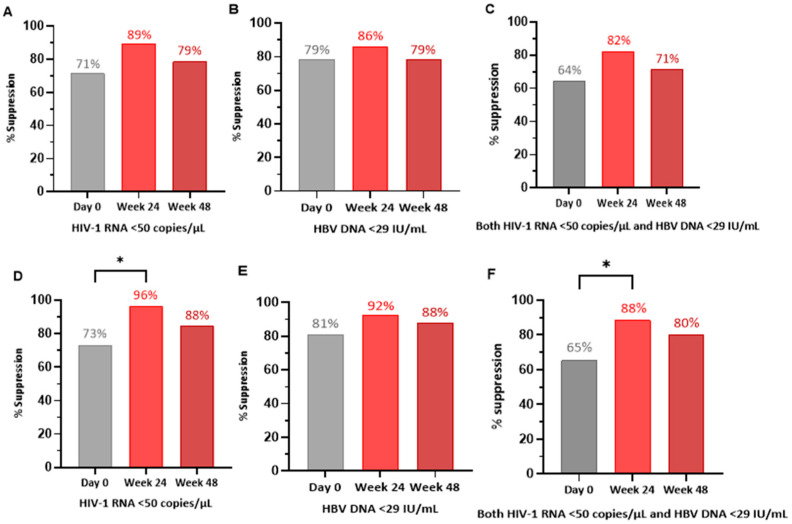
Primary and secondary efficacy results for the intention-to-treat population (N = 28) and the per-protocol population. (**A**–**C**) graphically illustrate the proportions of HIV-1 viral suppression (**A**), HBV viral suppression (**B**), and combined HIV-1 and HBV suppression (**C**) in the intention-to-treat (ITT) population. (**D**–**F**) graphically illustrate the proportions of HIV-1 viral suppression (**D**), HBV viral suppression (**E**), and combined HIV-1 and HBV suppression (**F**) in the per-protocol (PP) population. * indicates statistical significance at *p*-value < 0.05, as determined using two-tailed exact McNemar test. Legend: HIV-1—human immunodeficiency virus-1; HBV—hepatitis B virus; IU/mL—international units per milliliters.

A total of 28 participants received at least one dose of the drug under analysis and thus were included in the ITT population analysis (Table 2 and Figure 1A–C). At Week 24 (the primary efficacy timepoint), 25 (89%) had HIV-1 RNA < 50 copies/mL, and 24 (86%) had HBV DNA < 29 IU/mL. A total of 23 (82%) of the participants had both HIV-1 RNA < 50 copies/mL and HBV DNA < 29 IU/mL. At Week 48 (the secondary efficacy timepoint), 22 (79%) exhibited HIV-1 suppression, 22 (79%) exhibited HBV suppression, and 20 (71%) exhibited suppression for both. All four patients with abnormal ALT levels at baseline exhibited normalization of their ALT levels by Weeks 24 and 48.

Of the 20 participants who were HIV-suppressed at baseline (prior to the ART switch), 19 (95%) still had HIV < 50 copies/mL at Week 24, while the 1 remaining patient was lost to follow-up prior to the Week 24 visit (no data are available beyond the Day 1 visit), and 16 (80%) still had HIV < 50 copies/mL at Week 48. Three participants at Week 48 had detectable HIV RNA levels ranging from 58–340 copies/mL, which may represent a blip (usually <400); they all had consistent viral RNA levels of < 50 IU/mL prior to this last evaluation. Of the eight participants who were not HIV-suppressed at baseline, six (75%) achieved HIV < 50 copies/mL by Week 24, one achieved this endpoint by Week 48, and one was removed from study due to non-adherence even prior to the Week 12 visit. There were no relevant NRTI or INSTI resistance observed during follow-up visits.

Of the 22 participants who were HBV-suppressed at baseline, 21 (95%) still had HBV DNA < 29 IU/mL by Week 24, with the 1 remaining having been lost to follow-up prior to the Week 24 visit and presenting no HBV DNA data beyond Day 1. Of the six participants who were not HBV-suppressed at baseline, three (50%) achieved HBV DNA < 29 IU/mL at Week 24 and maintained this by Week 48; two (33%) did not achieve HBV < 29 IU/mL during the study period; and the remaining one was dismissed from the study due to non-adherence prior to the Week 12 visit. There were two participants who failed to achieve HBV < 29 IU/mL by Week 48: one had a 3.1 Log_10_ decline in their HBV DNA levels (compared to the baseline) at the end of the study; and the other reported episodes of missed doses at the Week 36 visit and had an HBV DNA value that was 0.64 Log_10_ higher at Week 48 compared to the baseline (6.16 vs. 5.52 Log_10_ IU/mL). No relevant resistance mutations were observed in those two who failed to achieve suppression. Additionally, all six participants who were not on HBV-active ART at baseline exhibited HBV DNA suppression at Weeks 24 and 48.

Of the 18 participants who were both HIV- and HBV-suppressed at baseline, 17 (94%) remained HIV- and HBV-suppressed at the Week 24 visit, while 1 was lost to follow-up, as noted above; 14 (78%) remained HIV- and HBV-suppressed at the Week 48 visit. Of the ten participants who were not both HIV- and HBV-suppressed at baseline, five (50%) exhibited HIV and HBV suppression at the Week 24 and Week 48 visits, one achieved such suppression by Week 24 but not did not exhibit it at Week 48, one exhibited such suppression at the Week 48 visit but not at the Week 24 visit, and two failed to achieve combined HIV-HBV suppression at both timepoints. One participant (as previously noted) was dismissed from this study due to noncompliance.

A total of 26 participants were included in the PP population for the primary endpoint analyses (Week 24), and 25 were included for the secondary endpoint analyses (Week 48) (Table 3 and Figure 1D–F). At Week 24, 25 of the participants (96%) had HIV-1 RNA < 50 copies/mL and 24 (92%) had HBV DNA < 29 IU/mL; 23 participants (88%) had both HIV-1 RNA < 50 copies/mL and HBV DNA < 29 IU/mL. At Week 48, 22 of the participants (88%) were HIV-1 suppressed, 22 (88%) were HBV-DNA-suppressed, and 20 (80%) exhibited suppression for both viruses. All four participants with abnormal ALT levels at baseline had normalized ALT levels at Weeks 24 and 48.

Analysis conducted using the nonlinear mixed model (NLMM) showed that the odds of achieving and/or maintaining HIV-1 RNA < 50 copies/mL in the ITT and PP populations from Day 0 to Week 48 tended to increase over time. However, these results were not statistically significant (OR = 1.26, 95%CI = (0.61, 2.60) and OR = 1.97, 95%CI = (0.65, 5.90), respectively) (Supplemental Table S2E). Similar results were also analyzed in regard to the odds of maintaining and/or achieving HBV DNA < 29 IU/mL and both HIV-1 RNA < 50 copies/mL and HBV DNA < 29 IU/mL in the ITT and PP populations, but no statistical significances were detected.

In the per-protocol population, among the six individuals who had HIV-1 RNA levels of ≥50 copies/mL on Day 0, all (100%) experienced a decrease to <50 copies/mL by Week 24. Conversely, there were no cases (0 out of 6) where HIV-1 RNA levels increased from <50 copies/mL on Day 0 to ≥50 copies/mL at the Week 24 visit. In other words, all discordant cases showed improvement, and none of them showed worsened conditions. In addition, among the six individuals who had both HIV-1 RNA ≥ 50 copies/mL and HBV DNA ≥ 29 IU/mL on Day 0, all (100%) experienced a decrease to HIV-1 RNA < 50 copies/mL and HBV DNA < 29 IU/mL by Week 24. Conversely, there were no cases (0 out of 6) where HIV-1 RNA and HBV DNA levels increased to ≥50 copies/mL and ≥29 IU/mL. These results suggest a significant treatment effect, as indicated by the *p*-value of 0.031 (*p* < 0.05) (Table 3 and Table S2B).

HBsAg data were available for 26 participants at the Week 24 visit and 25 participants at the Week 48 visit. Of those with available HBsAg results, none exhibited HBsAg loss, and none had anti-HB antibody seroconversion by Weeks 24 and 48. Eleven of the twenty-five (44%) participants were HBeAg-positive at baseline. Of those with available HBeAg results, none showed HBeAg loss at the Week 24 visit, but 2/11 (18%) exhibited HBeAg loss at the Week 48 visit. Neither of the two had anti-HBe seroconversion by Weeks 24 and 48.

The mean CD4 count and percentage were 569 cells/µL (median: 551, range: 149–1294, IQR: 317–749) and 31.5% (n = 25; median: 33.5, range: 10.4–49.4, IQR: 25.1–41.1), respectively, for the 25 participants with available data at the Week 24 visit, but neither were statistically different from the baseline (*p* = 0.35 and 0.31, respectively). However, the mean CD4 count and percentage at the Week 48 visit were numerically higher compared to the baseline but not statistically significant (*p* = 0.05 for both), with a mean CD4 count and percentage of 621 cells/µL (median: 640, range: 218–1284, IQR: 384–745) and 32.9% (median: 32.5, range: 12.8–50.3, IQR: 25.7–40.0), respectively.

Twenty of the twenty-one Baltimore-site-enrolled participants had Fibroscan test results by Week 24. Fourteen had Fibroscan results by Week 48. Although six of the remaining seven participants without fibrosis results attended their Week 48 (end of study) visit, Fibroscan was not conducted due to COVID-19 research restrictions limiting person-to-person contact. The mean fibrosis score for the Week 24 visit was 6.33 kPa (range: 3.4–25.4, IQR: 4.6–6.4), whereas that for Week 48 was 7.78 kPa (range: 3.3–27.0, IQR: 4.0–9.1). The declines in the mean fibrosis scores at both the Week 24 and 48 visits were not statistically significant.

### 3.3. Safety Results

Thirty-eight AEs were reported, of which eleven were related to the treatment. All these AEs were either mild or moderate in severity (Table 4). The most common AE, related or unrelated, was upper respiratory tract infection (5/28 or 18%). The most common treatment-related AE was nausea, described as mild and transient, in 2 of 28 (7%) participants. Other treatment-related AEs (one each) included abdominal pain, diarrhea, headache, irritability, night sweats, peripheral edema, rash, and vivid dreams. All treatment-related AEs were grade 1 (mild) in severity. No participants discontinued the treatment due to an AE. No serious AEs were reported. No hepatitis flares were observed.

## 4. Discussion

To the best of our knowledge, our study is the first BIC/FTC/TAF switch trial conducted on patients with HIV/HBV coinfection. Moreover, we enrolled a population with a unique but important demographic profile. Close to 90% were Black (African American or African-born), a historically underserved population in the United States. Almost 30% were migrants from Africa, and, in an unexpected finding, our study had a relatively high (14%) proportion of hepatitis D (HDV)-positive participants compared to the United States HBsAg-positive population of about 4.2% [14,15].

Numerically larger proportions of the participants were HIV- and HBV-suppressed by Week 24 compared to the baseline, though this did not reach statistical significance due to the small sample size. Nonetheless, if the two participants who were either noncompliant or lost to follow-up are excluded, combined HIV-1 and HBV suppression at Week 24 was significantly higher than that at baseline (88% vs. 65%, *p* = 0.031). At the end of the participation period (Week 48), the proportion of participants who were both HIV- and HBV-suppressed was higher (80% vs. 65%) than prior to the BIC/FTC/TAF switch, albeit not to a statistically significant degree. We think that improved adherence was a major contributor to the improved suppression rates observed in our study, but in the absence of a comparator, it would be difficult to tease out the relative contribution of improved adherence versus better drug efficacy in our study. As previously observed among HIV mono-infected patients in registrational trials [8,9] and, more recently, among HIV/HBV treatment-naïve patients in the ALLIANCE trial [10], BIC/FTC/TAF was well tolerated by the participants, with none of them discontinuing treatment due to AEs. There were also no SAEs, deaths, or hepatitis B flare ups observed while the patients were on BIC/FTC/TAF during the study.

Interestingly, ALLIANCE, a randomized, double-blind, active-controlled trial comparing BIC/FTC/TAF to DTG + FTC/TDF in treatment-naïve adults with HIV/HBV coinfection, found numerically greater rates of HBsAg loss (12.6% vs. 5.8%), HBsAg seroconversion (8.4% vs. 3.3%), and HBeAg loss (25.6% vs. 14.4%) by Week 48 for BIC/FTC/TAF versus DTG + F/TDF, respectively. Moreover, the HBeAg seroconversion rate at the Week 48 visit was significantly higher in the BIC/FTC/TAF arm (23.3% vs. 11.3%, *p* < 0.05) [10]. Although none of our treatment-experienced participants demonstrated HBsAg loss after 48 weeks of BIC/FTC/TAF therapy, two (18%) did show HBeAg loss without anti-HBe seroconversion by Week 48. Initiating ART has been observed to induce higher rates of HBsAg clearance in treatment-naïve HIV/HBV-coinfected patients compared to HBV mono-infected patients, which has been attributed to immune reconstitution helping to control HBV. Such immune reconstitution in treatment-naïve patients is unlikely to be seen in a switch study, where participants are treatment-experienced and likely to have already experienced immune reconstitution as a result of prior initiation of ART. Another possible explanation for the lack of HBsAg clearance is that the one-year follow-up period may have been too short a duration to detect this outcome measure in this treatment-experienced population.

Our study population was small and did not reach the planned sample size primarily because most treatment-experienced, coinfected individuals had already switched to BIC/FTC/TAF in our clinics. This study was also severely impacted by COVID-19 travel and research restrictions, particularly between March 2020 and August 2020, when screening and nonessential study visits were restricted. We were able to enroll 28 participants, slightly less than half of the target based on power calculation (n = 60), increasing the risk of Type 2 error and the failure to detect real differences due to the study being underpowered. Nonetheless, the data from this switch study add to and further strengthen the growing evidence supporting the use of BIC/FTC/TAF for the treatment of HIV/HBV-coinfected patients.

## 5. Conclusions

In summary, switching therapy to BIC/FTC/TAF was observed to be safe, well tolerated, and a suitable option for the treatment of adults coinfected with HIV and HBV, regardless of whether either virus was suppressed prior to the switch. We did not observe any HBsAg loss or seroconversion over 48 weeks in this treatment-experienced population. Nonetheless, we observed two participants who cleared HBeAg by Week 48 but had not seroconverted at this time. Already a preferred drug regimen and commonly prescribed for patients with HIV/HBV coinfection based on its efficacy, safety, simplicity, and high resistance barrier, our results further justify the use of BIC/FTC/TAF as a regimen of choice for this population.

## Figures and Tables

**Table 1:** Baseline demographics and disease characteristics.

	N	Percent
Total Enrolled (N)	28	-
Age, in years		
Mean (range)	50.1 (34–71)	
Sex		
Female	4	14
Male	24	86
Race		
Black or African American	25	89
White	3	11
Ethnicity		
Non-Hispanic	28	100
HIV RNA (copies/mL)		
<50	20	71
50–500	6	21
>500	2	7
CD4 Count (cells/µL)		
>500	12	43
200–500	15	54
<200	1	4
Duration of HIV suppression		
>12 months	19	68
6–12 months	1	4
<6 months	0	0
Not suppressed	8	29
HBV DNA < 29 IU/mL	22	79
qHBsAg (IU/mL)		
<10 (LLOQ)	5	18
10–100	4	14
101–1000	6	21
1001–10,000	4	14
>10,000	9	32
HBeAg positive (%) *	12	48
Anti-HBe positive (%) *	10	40
HCV antibody positive	5	18
HDV antibody positive	5	18
ALT, normal	24	86

* Data were not available for 3 participants. Legend: ALT: alanine aminotransferase; HBV: hepatitis B virus; HCV: hepatitis C virus; HDV: hepatitis D virus; qHBsAg: quantitative hepatitis B surface antigen; HBeAg: hepatitis B e antigen; anti-HBe: hepatitis B e antibody; LLOQ: lower limit of quantification; N: number of participants.

**Table 2:** Efficacy results for intention-to-treat population.

Endpoints	N	Percent	*p* Value *
Primary Efficacy			
Total number of participants with Week 24 data	28	-	
No. of participants for whom HIV-1 RNA < 50 copies/mL by Week 24	25	89%	0.125
No. of participants for whom HBV DNA < 29 IU/mL by Week 24	24	86%	0.625
No. of participants with both HIV-1 RNA < 50 copies/mL and HBV DNA < 29 IU/mL by Week 24	23	82%	0.125
Secondary Efficacy			
Total number of participants with Week 48 data	28	-	
No. of participants for whom HIV-1 RNA < 50 copies/mL by Week 48	22	79%	0.754
No. of participants for whom HBV DNA < 29 IU/mL by Week 48	22	79%	1.000
No. of participants with both HIV-1 RNA < 50 copies/mL and HBV DNA < 29 IU/mL at Week 48 visit	20	71%	0.754
Total no. of participants with abnormal ALT levels at baseline	4	-	
No. of participants with normalized ALT levels by Week 24	4	100%	
No. of participants with normalized ALT levels by Week 48	4	100%	

* Exact McNemar Test, two-tailed. R version 4.3.3. Full statistical analysis results can be found in Supplemental Table S2. N: number of participants.

**Table 3:** Primary and secondary efficacy results obtained for the per-protocol population.

Endpoints	N	Percent	*p* Value *
Primary Efficacy			
Total number of participants with Week 24 data	26	-	
No. of participants exhibiting HIV-1 RNA < 50 copies/mL at Week 24 visit	25	96%	0.031 **
No. of participants exhibiting HBV DNA < 29 IU/mL at Week 24 visit	24	92%	0.250
No. of participants with both HIV-1 RNA < 50 copies/mL and HBV DNA < 29 IU/mL at Week 24 visit	23	88%	0.031 **
Secondary Efficacy			
Total subjects with Week 48 data	25	-	
No. of participants exhibiting HIV-1 RNA < 50 copies/mL at Week 48 visit	22	88%	0.508
No. of participants exhibiting HBV DNA < 29 IU/mL at Week 48 visit	22	88%	1.000
No. of participants with both HIV-1 RNA < 50 copies/mL and HBV DNA < 29 IU/mL at Week 48	20	80%	0.508
Total number of participants with abnormal ALT levels at baseline	4	-	
No. of participants with normalized ALT levels at Week 24 visit	4	100%	
No. of participants with normalized ALT levels at Week 48 visit	4	100%	

* Exact McNemar test, two-tailed. R version 4.3.3. ** indicates statistical significance (i.e., *p* < 0.05). Complete statistical analysis results can be found in Supplemental Table S2. N: number of particiipants.

**Table 4:** Summary of treatment-emergent adverse events.

Item	N	Percent
Adverse Events Reported	38	-
Participants with at least 1 AE	16/28	57%
Participants with 2 or more AEs	5/28	18%
Participants with at least one treatment-related TEAE	7/28	25%
TEAE relationship		
Possibly, probably, or definitely related	11/38	29%
Not related	27/38	71%
TEAE Severity		
Grade 1—mild	37/38	97%
Grade 2—moderate	1/38	3%
Grade 3—severe	0/38	0%
Grade 4—potentially life-threatening	0/38	0%
TEAE leading to discontinuation of the drug	0	0%
Serious Adverse Events (SAEs) reported	0	0%
Participants with at least one SAE	0	0%
Participants with at least one treatment-related SAE	0	0%
Deaths	0	0%

AE: adverse event; TEAE: treatment-emergent adverse event; SAE: serious adverse event; N: number of participants.

## Data Availability

The data that support the findings of this study are available from the corresponding author upon reasonable request.

## Supplementary Materials

The following supporting information can be downloaded at https://www.mdpi.com/article/10.3390/v17040510/s1. Supplemental Figure S1. Consort diagram; Supplemental Table S1. Antiretroviral therapy prior to BIC/FTC/TAF switch. Supplemental Table S2: Statistical analysis of primary and secondary efficacy endpoints (S2A: efficacy results for ITT population; S2B: efficacy results for per-protocol (PP) population; S2C: efficacy results for per-protocol and ITT populations with 95% confidence interval; S2D: estimated proportion of change and 95% confidence interval; S2E: effects of time and age on the odds of outcomes).
