# Supplementary material for: Bictegravir/Emtricitabine/Tenofovir Alafenamide in Adults with HIV/HBV Coinfection: An Open-Label, Single-Arm, Safety and Efficacy Switch Study
Source: Viruses. 2025 Mar 31;17(4):510. doi: 10.3390/v17040510 (PMC12030933; doi:10.3390/v17040510)
Supplement: Supplementary file 1 [file viruses-17-00510-s001.zip › viruses-3514027-supplementary.pdf]

# Supplemental Figures and Tables:

### Supplemental Figure S1 – Consort Diagram

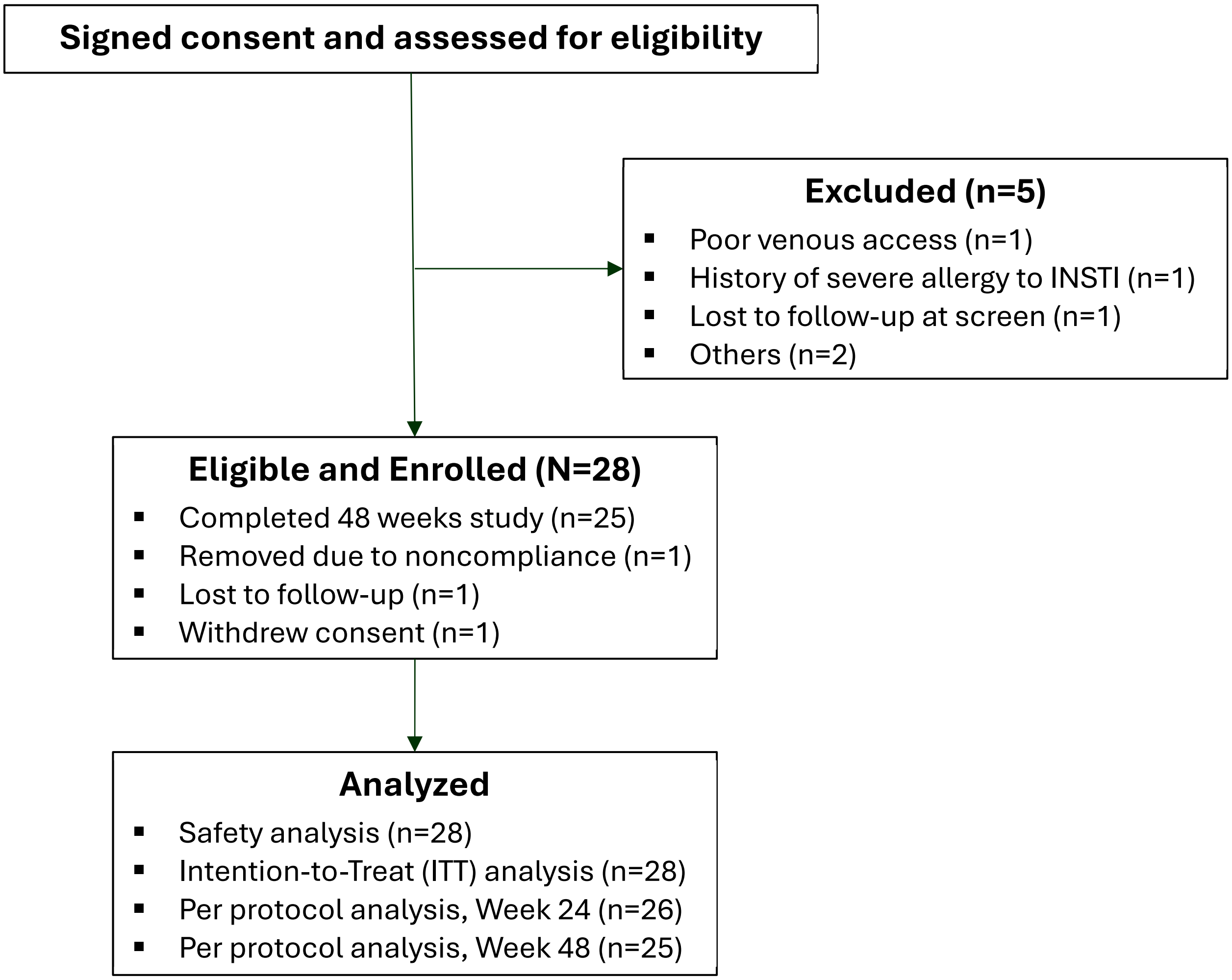


Legend: INSTI – integrase strand transfer inhibitor. INSTI – integrase strand transfer inhibitor; ITT – intention-to-treat.

**Supplemental Table S1. Antiretroviral therapy prior to B/F/TAF switch**

| Previous ART | N | % |
|---|---|---|
| Tenofovir-based regimen | 22 | 79% |
| Tenofovir alafenamide (TAF) | 19 | 68% |
| Tenofovir disoproxil fumarate (TDF) | 3 | 11% |
| Other NRTI | | |
| Abacavir (ABC) | 3 | 11% |
| Emtricitabine (FTC) | 22 | 79% |
| Lamivudine (3TC) | 3 | 11% |
| INSTI | 21 | 75% |
| Dolutegravir (DTC) | 13 | 46% |
| Elvitegravir (EVG), cobicistat-boosted | 8 | 29% |
| Protease Inhibitors | 5 | 18% |
| Atazanavir (ATV), boosted | 2 | 7% |
| Darunavir (DRV), boosted | 3 | 11% |
| NNRTI containing | 6 | 21% |
| Rilpivirine (RPV) | 5 | 18% |
| Efavirenz (EFV) | 1 | 4% |

INSTI: Integrase strand transfer inhibitor
NNRTI: Non-nucleos(t)ide reverse transcriptase inhibitor.
NRTI: Nucleos(t)ide reverse transcriptase inhibitor.

## Supplemental Table S2: Statistical Analysis of Primary and Secondary Efficacy Endpoints

### 2A: Efficacy Results for ITT Population

| Label | Endpoints | | | Total | P value* |
|---|---|---|---|---|---|
| **ITT-1** | **PRIMARY EFFICACY** | **Week 24** | | | |
| | **Day 0** | <50 | ≥50 | | 0.125 |
| | HIV-1 RNA <50 copies/mL | 19 | 1 | 20 | |
| | HIV-1 RNA ≥50 copies/mL | 6(0.86) † | 2 | 8 | |
| | **Total** | 25 | 3 | 28 | |
| **ITT-2** | **SECONDARY EFFICACY** | **Week 24** | | | |
| | **Day 0** | <29 | ≥29 | | 0.625 |
| | HBV DNA <29 IU/mL | 21 | 1 | 22 | |
| | HBV DNA ≥29 IU/mL | 3(0.75) † | 3 | 6 | |
| | **Total** | 24 | 4 | 28 | |
| | | **Week 24** | | | |
| **ITT-3** | **Day 0** | <50 & <29 | ≥50 & ≥29 | | 0.125 |
| | Both HIV-1 RNA <50 copies/mL and HBV DNA <29 IU/mL | 17 | 1 | 18 | |
| | Both HIV-1 RNA ≥50 copies/mL and HBV DNA ≥29 IU/mL | 6(0.86) † | 4 | 10 | |
| | **Total** | 23 | 5 | 28 | |
| | | **Week 48** | | | |
| **ITT-4** | **Day 0** | <50 | ≥50 | | 0.754 |
| | HIV-1 RNA <50 copies/mL | 16 | 4 | 20 | |
| | HIV-1 RNA ≥50 copies/mL | 6(0.6) † | 2 | 8 | |
| | **Total** | 22 | 6 | 28 | |
| | | **Week 48** | | | |
| **ITT-5** | **Day 0** | <29 | ≥29 | | 1.0 |
| | HBV DNA <29 IU/mL | 20 | 2 | 22 | |
| | HBV DNA ≥29 IU/mL | 2(0.5) † | 4 | 6 | |
| | **Total** | 22 | 6 | 28 | |
| | | **Week 48** | | | |
| **ITT-6** | **Day 0** | <50 & <29 | ≥50 & ≥29 | | 0.754 |
| | Both HIV-1 RNA <50 copies/mL and HBV DNA <29 IU/mL | 14 | 4 | 18 | |
| | Both HIV-1 RNA ≥50 copies/mL and HBV DNA ≥29 IU/mL | 6(0.6) † | 4 | 10 | |
| | **Total** | 20 | 8 | 28 | |

*Exact McNemar test, two-tailed. R version 4.3.3.
Total change is the sum of discordant cells (i.e., number of cases in which a change occurred).
†Proportion of change is the ‘one of discordant cells’ divided by the ‘total change’

## 2B: Efficacy Results for Per Protocol (PP) Population

| Label | Endpoints | | | Total | P value* |
|---|---|---|---|---|---|
| **PP-1** | **PRIMARY EFFICACY** | **Week 24** | | | |
| | **Day 0** | <50 | ≥50 | | 0.031** |
| | HIV-1 RNA <50 copies/mL | 19 | 0 | 19 | |
| | HIV-1 RNA ≥50 copies/mL | 6(1) † | 1 | 7 | |
| | **Total** | 25 | 1 | 26 | |
| **PP-2** | **SECONDARY EFFICACY** | **Week 24** | | | |
| | **Day 0** | <29 | ≥29 | | 0.25 |
| | HBV DNA <29 IU/mL | 21 | 0 | 21 | |
| | HBV DNA ≥29 IU/mL | 3(1) † | 2 | 5 | |
| | **Total** | 24 | 2 | 26 | |
| | | **Week 24** | | | |
| **PP-3** | **Day 0** | <50 & <29 | ≥50 & ≥29 | | 0.031** |
| | Both HIV-1 RNA <50 copies/mL and HBV DNA <29 IU/mL | 17 | 0 | 17 | |
| | Both HIV-1 RNA ≥50 copies/mL and HBV DNA ≥29 IU/mL | 6(1) † | 3 | 9 | |
| | **Total** | 23 | 3 | 26 | |
| | | **Week 48** | | | |
| **PP-4** | **Day 0** | <50 | ≥50 | | 0.508 |
| | HIV-1 RNA <50 copies/mL | 16 | 3 | 19 | |
| | HIV-1 RNA ≥50 copies/mL | 6(0.67) † | 0 | 6 | |
| | **Total** | 22 | 3 | 25 | |
| | | **Week 48** | | | |
| **PP-5** | **Day 0** | <29 | ≥29 | | 1.0 |
| | HBV DNA <29 IU/mL | 20 | 1 | 21 | |
| | HBV DNA ≥29 IU/mL | 2(0.67) † | 2 | 4 | |
| | **Total** | 22 | 3 | 25 | |
| | | **Week 48** | | | |
| **PP-6** | **Day 0** | <50 & <29 | ≥50 & ≥29 | | 0.508 |
| | Both HIV-1 RNA <50 copies/mL and HBV DNA <29 IU/mL | 14 | 3 | 18 | |
| | Both HIV-1 RNA ≥50 copies/mL and HBV DNA ≥29 IU/mL | 6(0.67) † | 2 | 10 | |
| | **Total** | 20 | 5 | 28 | |

*Exact McNemar test, two-tailed. R version 4.3.3. **Statistically significant (i.e., $p < 0.05$)
Total change is the sum of discordant cells (i.e., number of cases in which a change occurred).
†Proportion of change is the 'one of discordant cells' divided by the 'total change'

PP-1: The proportion of cases in which a change resulted in a specific outcome was estimated to be 100% (6/6) (i.e., the proportion that a day 0 measurement has HIV-1 RNA ≥50, while week 24 has HIV-1 RNA <50 is 1). This means that 100% of all trials experienced that change. The 95% confidence interval for this ratio is 54% to 100%,

representing a 95% chance that the true probability that a change will result in a particular outcome is within this range. And this result is statistically significant (p=0.031).

PP-3: The proportion of cases in which a change resulted in a specific outcome was estimated to be 100% (6/6) (i.e., the proportion that a day 0 measurement has HIV-1 RNA ≥50 and HBV DNA ≥29, while week 24 has HIV-1 RNA <50 and HBV DNA <29 is 1). This means that 100% of all trials experienced that change. The 95% confidence interval for this ratio is 54% to 100%, representing a 95% chance that the true probability that a change will result in a particular outcome is within this range. And this result is statistically significant (p=0.031).

## 2C: Efficacy Results using Per Protocol & ITT Population with 95% Confidence Interval

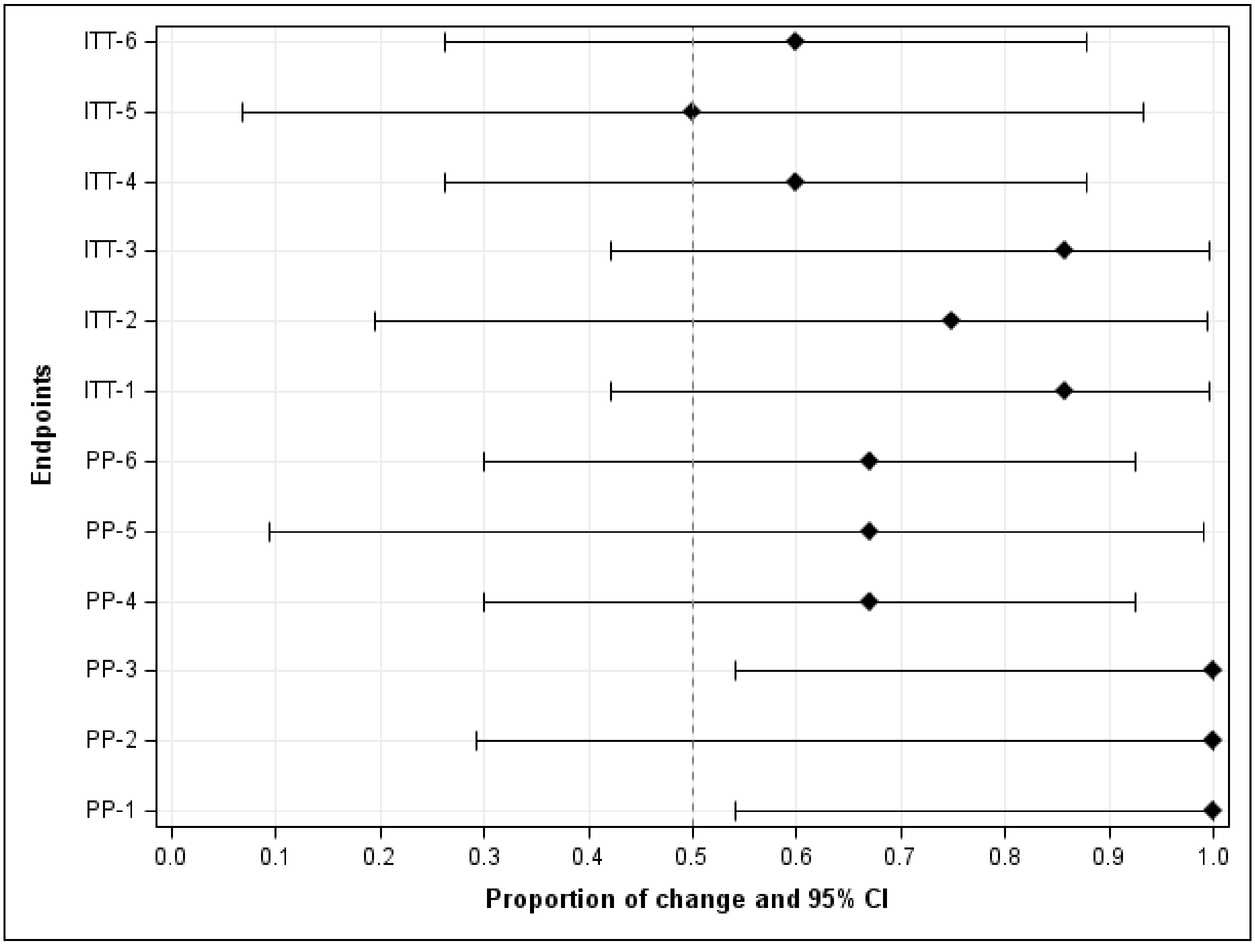


Alternative hypothesis is the proportion of change is not equal to 0.5

**2D: Estimate proportion of change and 95% confidence interval**

| Endpoints label | Proportion of change(95%CI) | p value |
|---|---|---|
| PP-1 | 1.00 (0.54 – 1.00) | 0.031 |
| PP-2 | 1.00 (0.29 – 1.00) | 0.250 |
| PP-3 | 1.00 (0.54 – 1.00) | 0.031 |
| PP-4 | 0.67 (0.29 – 0.93) | 0.508 |
| PP-5 | 0.67 (0.09 – 0.99) | 1.000 |
| PP-6 | 0.67 (0.29 – 0.93) | 0.508 |
| ITT-1 | 0.86 (0.42 – 0.99) | 0.125 |
| ITT-2 | 0.75 (0.19 – 0.99) | 0.625 |
| ITT-3 | 0.86 (0.42 – 0.99) | 0.125 |
| ITT-4 | 0.60 (0.26 – 0.88) | 0.754 |
| ITT-5 | 0.50 (0.06 – 0.94) | 1.000 |
| ITT-6 | 0.60 (0.26 – 0.88) | 0.754 |

**2E. Effects of time and age on the odds of outcome**

| Endpoints | Predictor | Odds ratio (95%CI) | p value |
|---|---|---|---|
| **ITT** | | | |
| HIV-1 RNA <50 copies/mL | Time point (baseline, week24, week48) | 1.26 (0.61, 2.60) | 0.530 |
| | Age | 1.05 (0.99, 1.12) | 0.084 |
| HBV DNA <29 IU/mL | Time point (baseline, week24, week48) | 1.00 (0.61, 1.62) | 1.000 |
| | Age | 1.07 (0.98, 1.16) | 0.124 |
| Both HIV-1 RNA <50 copies/mL and HBV DNA <29 IU/mL | Time point (baseline, week24, week48) | 1.21 (0.66, 2.22) | 0.529 |
| | Age | 1.06 (1.00, 1.14) | 0.046 |
| **PP** | | | |
| HIV-1 RNA <50 copies/mL | Time point (baseline, week24, week48) | 1.97 (0.65, 5.90) | 0.228 |
| | Age | 1.06 (0.99, 1.13) | 0.091 |
| HBV DNA <29 IU/mL | Time point (baseline, week24, week48) | 1.36 (0.67, 2.76) | 0.392 |
| | Age | 1.09 (0.98, 1.21) | 0.104 |
| Both HIV-1 RNA <50 copies/mL and HBV DNA <29 IU/mL | Time point (baseline, week24, week48) | 1.59 (0.72, 3.45) | 0.245 |
| | Age | 1.07 (0.99, 1.16) | 0.052 |

Nonlinear mixed model was applied
